# An Applied Research Partnership to Improve The Children’s Behavioral Health System Of Care

**DOI:** 10.23889/ijpds.v9i5.2586

**Published:** 2024-09-18

**Authors:** Sharon Zanti

**Affiliations:** 1 Actionable Intelligence for Social Policy; 2 University of Pennsylvania

## Objective and Approach

This session describes a doctoral student-led process to align research, policy, and practice partners around the use of integrated data to address a policy priority—the mounting youth mental health crisis. The project partners engaged in a five-year governance process to leverage integrated administrative data from a large health and human service agency in one U.S. state. Qualitative field notes—collected while conducting quantitative analyses and engaging partners—were analyzed to document a realistic timeline and process for ethical, actionable research using cross-sector data.

## Results

Five key lessons for partnership-engaged research emerged, emphasizing the importance of adapting to changes in project themes, leveraging strong partnership to address data quality concerns, nurturing existing and new relationships, balancing data access and data privacy, and supplementing administrative data with contextual data.

## Conclusions

Though engaging deeply with partners takes substantial time and energy, there are benefits to researchers, policymakers, practitioners, and most importantly, residents whose data are represented. Furthermore, building pathways to legally and ethically access integrated data can promote the efficient, strategic use of data for social policy research, and ultimately, be leveraged to support evidence-based policymaking in an ever-changing world.

## Implications

This session underscores the value of forming applied research partnerships and building legal and ethical pathways to access integrated data when tackling complex social policy issues.

